# Toward Telemonitoring in Immune-Mediated Inflammatory Diseases: Protocol for a Mixed Attention Model Study

**DOI:** 10.2196/55829

**Published:** 2024-04-22

**Authors:** Marta Novella-Navarro, Jose M Iniesta-Chamorro, Diego Benavent, Javier Bachiller-Corral, Enrique Calvo-Aranda, Helena Borrell, Laura Berbel-Arcobé, Victoria Navarro-Compan, Xabier Michelena, Leticia Lojo-Oliveira, Jaime Arroyo-Palomo, Mariana Diaz-Almiron, Verónica García García, Irene Monjo-Henry, Claudia María Gómez González, Enrique J Gomez, Alejandro Balsa, Chamaida Plasencia-Rodríguez

**Affiliations:** 1 Rheumatology Department Hospital Universitario La Paz Madrid Spain; 2 Biomedical Engineering and Telemedicine Centre Center for Biomedical Technology Escuela Tecnica Superior de Ingenieros de Telecomunicacion, Universidad Politecnica de Madrid Madrid Spain; 3 Rheumatolgy Department Hospital Universitari Bellvitge Barcelona Spain; 4 Rheumatology Department Hospital Universitario Ramon y Cajal Madrid Spain; 5 Rheumatology Department Hospital Universitario Infanta Leonor Madrid Spain; 6 Hospital Universitari Vall D´Hebron Barcelona Spain; 7 Biostatistics Hospital Universitario La Paz Madrid Spain; 8 Centro De Investigación Biomédica En Red De Bioingeniería, Biomateriales Y Nanomedicina Madrid Spain

**Keywords:** digital health, mHealth, telemonitoring, rheumatic musculoskeletal diseases, digital resources, mixed attention model, rheumatic disease, musculoskeletal diseases, chronic diseases, pain, inflammation, antirheumatic drugs, telemonitoring, rheumatology, hybrid care model, care model, MAM, implementation, clinical outcome

## Abstract

**Background:**

Rheumatic and musculoskeletal diseases (RMDs) are chronic diseases that may alternate between asymptomatic periods and flares. These conditions require complex treatments and close monitoring by rheumatologists to mitigate their effects and improve the patient’s quality of life. Often, delays in outpatient consultations or the patient’s difficulties in keeping appointments make such close follow-up challenging. For this reason, it is very important to have open communication between patients and health professionals. In this context, implementing telemonitoring in the field of rheumatology has great potential, as it can facilitate the close monitoring of patients with RMDs. The use of these tools helps patients self-manage certain aspects of their disease. This could result in fewer visits to emergency departments and consultations, as well as enable better therapeutic compliance and identification of issues that would otherwise go unnoticed.

**Objective:**

The main objective of this study is to evaluate the implementation of a hybrid care model called the mixed attention model (MAM) in clinical practice and determine whether its implementation improves clinical outcomes compared to conventional follow-up.

**Methods:**

This is a multicenter prospective observational study involving 360 patients with rheumatoid arthritis (RA) and spondylarthritis (SpA) from 5 Spanish hospitals. The patients will be followed up by the MAM protocol, which is a care model that incorporates a digital tool consisting of a mobile app that patients can use at home and professionals can review asynchronously to detect incidents and follow patients' clinical evolution between face-to-face visits. Another group of patients, whose follow-up will be conducted in accordance with a traditional face-to-face care model, will be assessed as the control group. Sociodemographic characteristics, treatments, laboratory parameters, assessment of tender and swollen joints, visual analog scale for pain, and electronic patient-reported outcome (ePRO) reports will be collected for all participants. In the MAM group, these items will be self-assessed via both the mobile app and during face-to-face visits with the rheumatologist, who will do the same for patients included in the traditional care model. The patients will be able to report any incidence related to their disease or treatment through the mobile app.

**Results:**

Participant recruitment began in March 2024 and will continue until December 2024. The follow-up period will be extended by 12 months for all patients. Data collection and analysis are scheduled for completion in December 2025.

**Conclusions:**

This paper aims to provide a detailed description of the development and implementation of a digital solution, specifically an MAM. The goal is to achieve significant economic and psychosocial impact within our health care system by enhancing control over RMDs.

**Trial Registration:**

ClinicalTrials.gov NCT06273306; https://clinicaltrials.gov/ct2/show/NCT06273306

**International Registered Report Identifier (IRRID):**

PRR1-10.2196/55829

## Introduction

### Background

Rheumatic and musculoskeletal diseases (RMDs) are a heterogeneous group of chronic diseases that mainly affect the joints, bones, muscles, tendons, and ligaments, but they can also affect other organs and systems. The growing significance of these diseases stems from their prevalence and their profound impact on the patient’s quality of life. Some examples of RMD are rheumatoid arthritis (RA), with a global prevalence estimated at 0.46% (95% CI 0.39-0.54), and spondyloarthritis (SpA), with a prevalence between 0.2% and 1.6% [1,2].

These diseases are characterized by symptoms including pain or inflammation that may flare up, requiring professional evaluation and treatment modifications. Because these flares may negatively impact a patient's quality of life, it is essential to identify them and act as early as possible. For this reason, closely monitoring the disease and its treatment is important. Sometimes, this follow-up can be hampered by the enormous burden of care and consultation time limitations typical of outpatient settings, as well as by the patients' difficulty in keeping appointments due, in many cases, to work-related reasons or displacements [3]. In addition, the complexity of the treatments administered can lead to doubts regarding dosage and potential contraindications or adverse effects [4-7]. For all these reasons, it is important to maintain open communication channels between patients and professionals to keep everyone informed of incidents in the evolution of the disease and treatment. Telemedicine and digital health thus present a novel opportunity for the clinical management of chronic patients. In this context, implementing telemonitoring in the field of rheumatology has great potential, as it can enable the close monitoring of patients with RMDs [8].

Telemedicine has seen widespread implementation in recent years, not only in rheumatology (as we want to highlight in this protocol) but also in other areas of health care (eg, emergency departments, intensive care units, pharmacies, primary care, etc) [9]. This approach to patient care is endorsed by the World Health Organization (WHO) [10], as increasing scientific evidence shows that it can be a very important channel in patient-physician communication, improving clinical care and aiding in therapeutic decision-making. In fact, it has gained more strength following the COVID pandemic, a time when face-to-face visits to hospitals or health centers were restricted, especially for patients who were more susceptible due to their clinical history [11,12]. Moreover, recently published literature on telemedicine in the context of rheumatology care suggests that telemedicine may be an effective mode of care delivery for diagnosing and managing rheumatic diseases. However, despite these promising results, more studies are still needed to evaluate the effectiveness of this new approach [13,14].

Telemedicine does not necessarily substitute the traditional care model but rather complements it. Both telemedicine and traditional care have their own advantages, and the most effective approach often involves integrating these models to provide comprehensive and patient-centered health care. Telemedicine can complement traditional care by offering additional options for delivering health care services. It provides a means for remote consultations, monitoring, and follow-up care, especially in situations where physical presence is not mandatory. However, for some cases, traditional care models with in-person consultations, diagnostic tests, and procedures may be more appropriate. Therefore, adopting hybrid models that combine in-person and telemedicine services may optimize the strengths of both models and provide a more flexible and adaptive health care system [15,16].

Considering the increasing relevance of telemedicine and the need to continue to provide further evidence, the objectives of this study are outlined in the following section.

### Objectives

The main objective is to evaluate the implementation of a conceptual framework for a hybrid care model called the mixed attention model (MAM) for use in clinical practice.

The secondary objectives are as follows: (1) to identify features associated with adherence to follow-up through the MAM; (2) to evaluate the technological acceptance of the digital solutions included in the MAM; (3) to assess the agreement between patient-reported join assessments and clinician-based join assessments; (4) to evaluate whether the implementation of the MAM improves clinical outcomes compared to conventional follow-up; and (5) to assess patients´ health literacy using a digital interface.

## Methods

### Study Context

Using this framework, we conducted a previous pilot study (Digireuma study) [17] on the needs of patients with RMDs to develop a treatment strategy combining face-to-face visits with telematics follow-up through a mobile health (mHealth) tool. This tool allowed for the monitoring of disease activity, functionality, and overall patient health using electronic patient-reported outcomes (ePROs). Moreover, patients could also report incidents such as flares between their outpatient visits. This pilot study essentially sought to identify unmet needs in the follow-up and treatment of patients with RMDs and redefine the functionalities and features of future versions of this digital tool. During a 3-month follow-up period, patients had the opportunity to complete disease-specific ePROs at a prespecified frequency, as well as flare-ups and medication changes at any time. The number of interactions and alerts was assessed. The usability of the mobile solution was measured using the Net Promoter Score (NPS). Following the MAM development, there were 46 patients, 22 with RA (48%) and 24 (52%) with SpA). There were a total of 4019 interactions in the RA group and 3160 in the SpA group. A total of 15 patients generated a total of 26 alerts, of which the majority were managed remotely. In terms of patient satisfaction, 65% of respondents were considered to have approved the mobile solution, resulting in an NPS of 57 and an overall rating of 4.3 out of 5 stars, so we conclude that the use of the digital health solution is feasible in clinical practice to monitor ePROs for RA and SpA. Therefore, these results highlighted the next steps in implementing this telemonitoring method in a multicenter setting.

Accordingly, with the results and feedback obtained from previous experience, we detail the design of a clinical study wherein we will evaluate a new version of our digital health solution for monitoring and treating patients with RA and SpA.

### MAM Explanation

**Figure 1:**
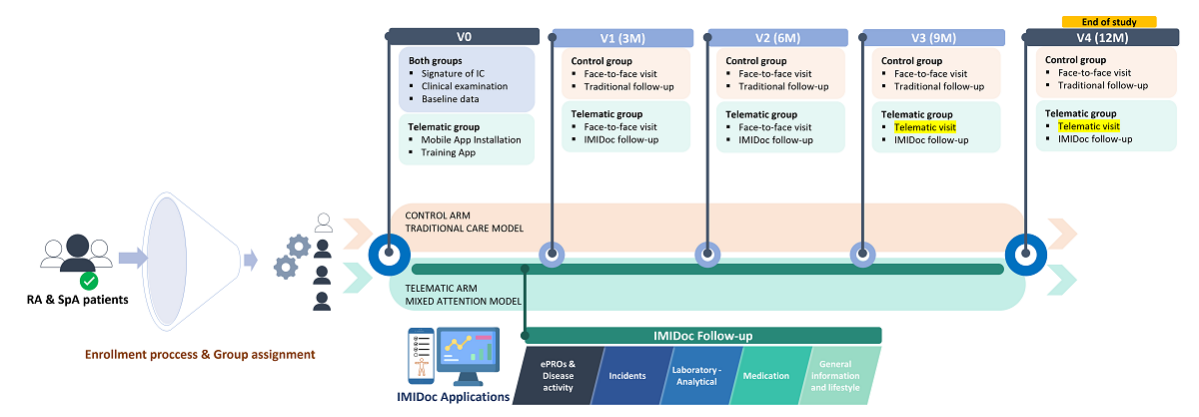
Flowchart describing the inclusion in the study and the different steps according to the timetable established for the face-to-face and digital visits. ePRO: electronic patient-reported outcome; IC: informed consent; RA: rheumatoid arthritis; SpA: spondyloarthritis.

All patients with RMDs treated with biological or targeted disease-modifying antirheumatic drugs (b/ts-DMARDs) are followed up in the immune-mediated disease (IMID) unit. This is a structured care unit comprising a multidisciplinary team of rheumatologists and specialized nurses. All care actions are protocolized, and face-to-face IMID unit visits of patients with RMDS under b/ts-DMARDs are recorded in a database. Based on the objectives described, a theoretical protocol was drawn up on how to implement a mixed model combining face-to-face visits with telematic follow-up. The MAM protocol [17] is a care model that incorporates a digital mobile app that patients can use at home and professionals can review asynchronously to detect incidents and monitor clinical evolution between face-to-face visits (Figure 1 depicts a flowchart escribing the participant inclusion in the study and subsequent steps). This protocol considers the role of each of the professionals in the unit, how face-to-face and telematic follow-up is carried out, and how to act according to the response to prescribed treatment.

### Development of the Digital Health Solution

A survey of health professionals and patients was carried out with the aim of identifying their needs and preferences regarding digital health as applied to rheumatology. Questions queried patients' familiarity with telemonitoring tools and mobile apps, the ease with which they could contact their rheumatologists, and their preferences and suggestions about the features that a digital solution should include. As for the professionals, they responded to a survey on the use of telemonitoring tools in clinical practice, training in the subject, and restrictions that hinder a wider implementation of telemedicine.

**Figure 2:**
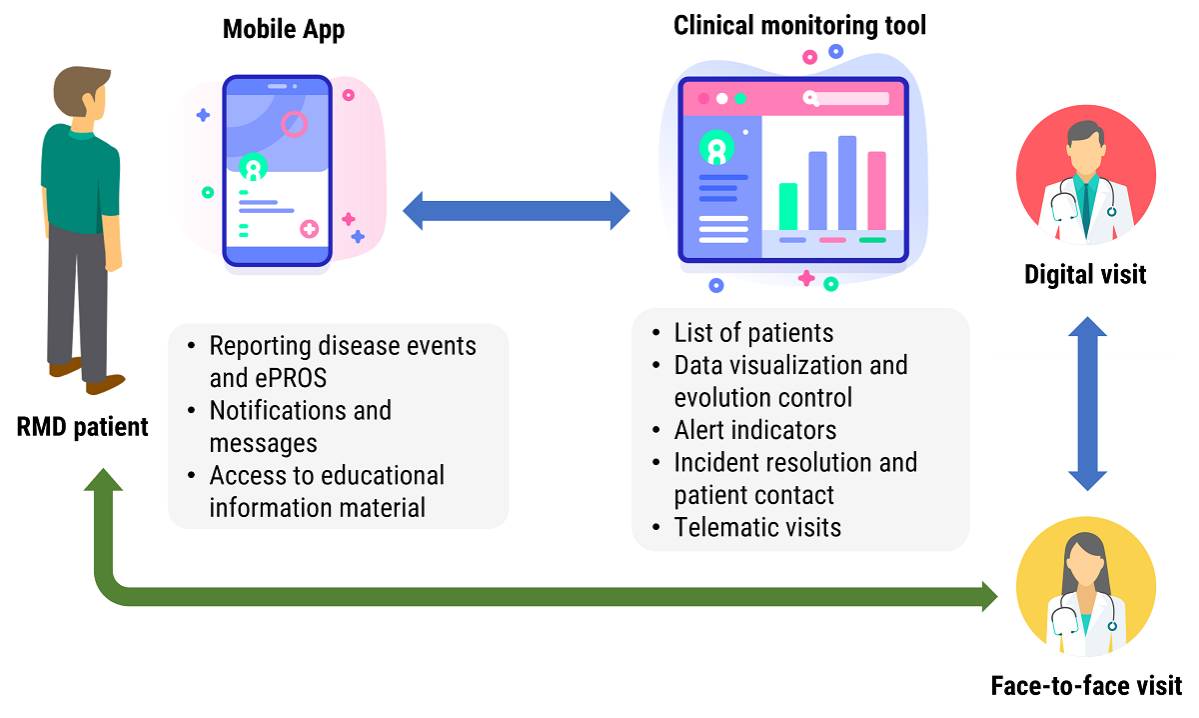
Digital health solution outline. ePRO: electronic patient-reported outcome; RMD: rheumatic and musculoskeletal disease.

Combining this information and following a team-based approach, the IMIDoc digital health tool was then designed and developed (Figure 2 shows the digital solution outline). Throughout the process, clinical, technical, and biomedical researchers and patient teams participated in a collaborative and interdisciplinary manner. IMIDoc is based on the MAM.

The IMIDoc technical platform consists of a mobile app for patients with RA and SpA, a web tool for clinicians, and the back-end infrastructure for data processing and storage. The IMIDoc platform has its own infrastructure to function independently of the hospital information system. Its back-end implementation is deployed in the cloud with a service-based technology approach that provides support to the IMIDoc mobile app and clinical tool.

The IMIDoc mobile app has been developed for both Android and iOS platforms and will be available through Google Play and the Apple Store, respectively. This implementation ensures that the app is easily accessible to a wide population of patients and accommodates various smart device preferences. On the other hand, the clinical tool is a web-based application accessible via a link in any standard web browser.

Patients with RA and SpA can record disease-related events and incidents, as well as ePROs, and they can receive messages from rheumatologists and automatic notifications regarding pending tasks and educational content. Likewise, rheumatologists can access the information recorded by patients through a web application. This enables clinicians to monitor and track the disease and contact patients to address any issues remotely.

### Study Design

#### Overview

This is a multicenter prospective observational study involving 360 patients with RA and SpA from 5 Spanish Hospitals: Hospital Universitario La Paz (HULP), Hospital Universitario Vall d´Hebron (HUVH), Hospital Universitario Bellvitge (HUB), Hospital Universitario Infanta Leonor (HUIL), and Hospital Universitario Ramón y Cajal (HURyC).

#### Participants

A total of 360 patients will be enrolled in the clinical study, comprising the group receiving MAM intervention and the control group, following the traditional care model matched by age and sex (Table 1). The inclusion criteria for patients in the MAM group are as follows: (1) adult patients >18 years; (2) patients with RA or SpA under treatment with b/tsDMARDs followed in the complex therapy unit (CTU); and (3) patients with an available smartphone. The exclusion criterion includes patients with conditions that hinder or prevent the use of a mobile app (eg, blindness, developmental delay, dementia, and digital illiteracy).

**Table 1:** Patients at each hospital treated with the MAM^a^ or a traditional care model.

Hospital	Patients in the MAM (n=280), n			Patients in the traditional care model (n=80), n	
	RA^b^	SpA^c^	RA		SpA
HULP^d^	40	40	10		10
HURyC^e^	30	30	10		10
HUVH^f^	30	30	10		10
HUIL^g^	20	20	5		5
HUB^h^	20	20	5		5

^a^MAM: mixed attention model.

^b^RA: rheumatoid arthritis.

^c^SpA: spondylarthritis.

^d^HULP: Hospital Universitario La Paz.

^e^HURyC: Hospital Universitario Ramón y Cajal.

^f^HUVH: Hospital Universitario Vall.

^g^HUIL: Hospital Universitario Infanta Leonor.

^h^HUB: Hospital Universitario Bellvitge.

All patients from outpatient consultations treated with b/tsDMARDs for at least 6 months will be invited to participate in the study. Those who have been invited but have declined will be registered, and then if they accept, will be enrolled in the study. The control group will be formed by patients with the same treatments and similar sociodemographic and clinical characteristics.

Due to the exploratory nature of the trial and the fact that the objectives will be measured with different end points, no formal sample size calculation was performed. Based on previous studies in rheumatology comparing clinical parameters using telemonitoring versus conventional monitoring, the number of patients included varied in a wide range (from 44 to 294 patients) [18-20]. The primary end point considered in our study in terms of a 20% reduction in face-to-face visits has not been previously studied, and there is no data in the literature to support the inclusion of a specific number of patients. Therefore, using a crude calculation, we estimated a sample size of 300 patients to be sufficient, considering an infinite population, a confidence level of 95%, a precision of 3%, and an assumption of 15% loss to follow-up. Furthermore, other important aspects to consider in telemonitoring projects with digital health tools are usability and feasibility. Recent studies, similar in objectives to the one proposed here, have included between 40 and 120 patients to evaluate the usability and feasibility of telemedicine applications and systems in RMDs [21]. As for the usability evaluation of the clinical tool discussed in this paper, the study is designed as a multicenter initiative, anticipating the participation of at least 10 health care professionals or digital clinicians. Considering the factors mentioned earlier and the expertise of these users within the field, we contend that this participant count is sufficient for the intended evaluation.

### Data Collection and Statistical Analysis

#### Data Sources

The application of MAM, via the use of IMIDoc incorporates 2 primary sources of information.

First, demographic, laboratory, clinical, and treatment characteristics will be extracted from electronic clinical records during the initial visit.

Second, ePROs recorded by patients participating in the IMIDoc users' arm through the application, as well as those documented by health care personnel during telematic and face-to-face visits through the web application, will be stored in the system's database in a pseudoanonymized and encrypted format.

Access to this data will be restricted exclusively to members of the research team. Data security will be supervised by the data protection officer. All actions performed on the platform will be traceable to the authorized personnel with access to the platform. User authentication tied to role-based security protocols will allow for the effective management of varying access privileges among members of the research team.

#### Data Collection and Schedule for Digital and Face-to-Face Visits

##### Overview

To facilitate understanding, variables are divided into general variables, face-to-face variables, and digital variables (Table 2). General variables correspond to background information captured during baseline visits. Face-to-face variables are captured during in-person visits and are registered in the electronic health record.

**Table 2:** Variables reported in both face-to-face and digital visits.

Dimensions and variables		AR^a^	SpA^b^	Frequency
**ePROs^c^ and disease activity**				
	PGA^d^, VAS^e^, TJC^f^, SJC^g^	✓	✓	Every 15 days
	Enthesis		✓	Every 15 days
	DAS28^h^, HAQ^i^	✓		Monthly
	ASAS-HI^j^, ASDAS^k^, BASDAI^l^		✓	Monthly
**Incidents**				
	Flares, infections, medication problems, and others	✓	✓	By occurrence
**Laboratory and analytical variables**				
	CRP^m^ and ESR^n^	✓	✓	Monthly
**Medication**				
	Medication list and reminders	✓	✓	On demand
	CQR^o^	✓	✓	Monthly
General information about the disease and lifestyle		✓	✓	Every 7 days

^a^RA: rheumatoid arthritis.

^b^SpA: spondylarthritis.

^c^ePRO: electronic patient-reported outcome.

^d^PGA: Patient Global Assessment.

^e^VAS: visual analog scale.

^f^TJC: tender joint count.

^g^SJC: swollen joint count.

^h^DAS28: Disease Activity Score 28.

^i^HAQ: Health Assessment Questionnaire.

^j^ASAS-HI: Assessment of Spondyloarthritis International Society Health Index.

^k^ASDAS: Ankylosing Spondylitis Disease Activity Score.

^l^BASDAI: Bath Ankylosing Spondylitis Disease Activity Index.

^m^CRP: C-reactive protein.

^n^ESR: erythrocyte sedimentation rate.

^o^CQR: Compliance Questionnaire for Rheumatology.

##### General Variables

General variables are those that correspond to clinical records documenting background or baseline information. The following will be collected during baseline visits: demographic variables (sex, age, and BMI); smoking habit; date of diagnosis; serological status for RA, such as rheumatoid factor (RF) anticitrullinated peptide antibodies (ACPA), and histocompatibility antigen (HLA)-B27 for SpA; and treatment information, including start date of current b/tsDMARDs and concomitant therapies such as prednisone, conventional disease-modifying antirheumatic drugs (csDMARDs), and previous treatments.

##### Face-to-Face Variables

Clinical activity and functional capacity will be evaluated according to specific indexes and questionnaires compiled in clinical practice.

For patients with RA, these include the tender joint count (TJC) and swollen joint count (SJC), Disease Activity Score 28 (DAS28), Simplified Disease Activity Score (SDAI), and Health Assessment Questionnaire (HAQ).

For patients with SpA, these include TJC and SJC, Ankylosing Spondylitis Disease Activity Score (ASDAS), Bath Ankylosing Spondylitis Disease Activity Index (BASDAI), Bath Ankylosing Spondylitis Functional Index (BASFI), and Assessment of Spondyloarthritis International Society Health Index (ASAS-HI).

Results from laboratory parameters, such as erythrocyte sedimentation rate (ESR) and C-reactive protein (CRP), are routinely determined in routine clinical practice at each patient visit.

##### Digital Variables and ePROS

For RA patients, the variables include self-assessed tender joint count (sTJC), self-assessed swollen joint count (sSJC), and SDAI. DAS28 will be calculated and managed as patient-derived DAS28 (PtDAS28) and HAQ. Finally, in patients with SpA, the variables include sTJC, sSJC, BASDAI, BASFI, and ASAS-HI.

Health care resource utilization will be documented, including the number of outpatient consultations, emergency visits, drug switches, and nursing and consultant calls. Questions for assessing patient status and treatment tolerance will be recorded. These will include questions such as, “Are you feeling well with the current treatment regimen?” “Have you had symptoms similar to previous outbreaks of your disease?” “If yes, how many outbreaks have you had during the last month?” “Have you experienced any symptoms that you attribute to tolerance problems with the current treatment?” “If yes, please indicate which symptom and to which treatment you attribute it.” “Have you had any recent fever or infections?” “Have you engaged in any physical activity equivalent to walking for 30 min at least 3 times during the past week?”

To assess the usability of the IMIDoc, questionnaires will collect information regarding willingness, such as the NPS, which evaluates how participants recommend the IMIDo, and follow-up with other patients using the MAM. The System Usability Scale (SUS), consisting of a 10-item questionnaire answered on a scale from 1 to 5 (strongly disagree to strongly agree), will also be used to assess the IMIDoc.

All patients who sign the informed consent form for this project will be invited to participate in a mixed clinical follow-up combining traditional face-to-face visits and home monitoring. The patients will be provided with access to a mobile app that can be downloaded free of charge and that was specially designed in consultation with and under supervision by rheumatologists. The patients will have access to complete questionnaires addressing disease activity, disability, and other domains such as ePROs (Figures 1 and 2). Face-to-face visits will be scheduled at baseline and 6 and 12 months, both for patients included in MAM and for those under the traditional care model.

### Support Resources for Patients

Explanatory material will be provided at the scheduled routine medical visit for all patients included. This explanatory material includes the information required to download the digital solution, the content, and recommendations regarding the frequency with which patients should fill out the questionnaires. Once the patient agrees to be part of the MAM, they will receive a phone call from the digitally partnered clinician to ensure understanding of all the material, clarify doubts, and resolve any issues.

Clinical activity, functional capacity, and overall health will be evaluated according to specific indexes and questionnaires included in IMIDoc, with a predefined frequency for each instrument (alternate assessments of pooled instruments every 15 days, implying a weekly assessment). Reminders with predefined frequencies for each variable will be sent to guarantee their fulfillment.

A messaging and alert system will be available whereby patients can notify their physician of any incidents.

Additionally, the IMIDoc educational material will be provided to patients in a short text format containing information of interest regarding their disease, the different treatments available, and explanatory videos recorded by collaborating rheumatologists.

### Workshops and Tasks for Collaborators

The established collaboration between HULP, HUVH, HUB, HUIL, and HRyC will also act as a facilitator for the development of this study. In fact, web-based meetings have already been held between researchers from all centers to evaluate the resources and the viability of implementing this project. Prior to starting patient recruitment, a web-based meeting will be held with the entire research team to discuss the best way to identify patients and carefully review the methodology that will be followed in the participating centers. Thus, all the information provided to patients will be shared and agreed upon by the centers. Moreover, reminders will be sent to the people responsible for and involved in each project task. In this regard, the clinician in charge of the telematic (digital clinicians) and clinical (onsite clinicians) for each center will be assigned. The incidents recorded in the tool will be periodically reviewed on a web interface by a physician whose clinical work is partly dedicated to this purpose (digital clinician). This professional or another professional from the CTU or biologic clinic (onsite clinician) will contact the patient by telephone to resolve the reported incident. The incident may be resolved completely remotely, or a face-to-face visit may be arranged to address the problem.

### Assessment of Primary and Secondary End Points:

The primary end point will be an at least 20% reduction in face-to-face visits compared to visits carried out in the previous year. This reduction in face-to-face visits will be driven by the number of incidents that can be resolved telematically, the number of total interactions, and the number of ePROS that patients complete during the follow-up period that allows us to assess adherence and telematic monitoring.

The secondary end points will be assessed by tracking the number of accesses and time per access to the digital solution per patient to assess adherence. Agreements between the patients' self-assessment and clinician assessment will be assessed to compare face-to-face visits at 6-month intervals with the closest assessment in the digital solution. We will also measure the proportion of patients in remission or low disease activity according to the activity index every 6 months. Usability and satisfaction perceived by patients will be assessed by the SUS NPS.

### Data Analysis

A descriptive analysis of the demographic and clinical variables will be performed. The results will be expressed as the mean and SD or median and IQR for continuous variables and absolute numbers and relative frequencies for categorical variables. The frequency data will be compared using a chi-square or Fisher exact test depending on the distribution of the variables. Comparisons for unpaired continuous data will be assessed using a Mann-Whitney U test or *t* test depending on the variable distribution. Comparisons of paired continuous data will be conducted using the paired *t* test or Wilcoxon test, depending on data distribution. *P*<.05 will be considered statistically significant. Univariate analysis will be carried out using the most relevant demographic and clinical variables (ie, age, sex, concomitant methotrexate, baseline activity score measured by DAS28 or BASDAI, disease duration, and smoking habit). In the multivariate analysis, only the variables with a *P*<0.1 in the univariate analysis will be included. Interactions and collinearity will be evaluated. The last phase of the study will consist of exploring the construction of a predictive model from the follow-up data using the MAM. For this purpose, artificial intelligence tools and techniques such as machine learning will be utilized. R-Cran (version 3.5.1; R Project for Statistical Computing) )software will be used for the statistical analysis.

### Ethical Considerations

This study will be conducted in accordance with the Declaration of Helsinki and the General Data Protection Regulation (GDPR) of the European Union. The protocol has been approved by the local Ethics Committees of HULP (PI-4519), HUB, HUIL, HRyC, and HUVH. This protocol was submitted to a public call for applications and obtained funding for the development of a solution and implementation of the MAM in clinical practice. Informed consent will be obtained from all participants prior to study onset. This study will have a protected and anonymized database, wherein data will be stored at baseline and throughout follow-up. All patient data will be collected and handled confidentially in a protected database located on an encrypted server owned by the digital health company in compliance with the European GDPR of May 25, 2018.

## Results

Recruitment began in March 2024 through initial contacts with patients at the IMID unit, and it will continue until December 2024. The follow-up period will be extended for 12 months for all patients. Data collection and analysis are scheduled for completion in December 2025. The main contribution of this protocol paper is a detailed description of the development of a digital solution to improve and implement an MAM in the field of RMDs.

## Discussion

We have designed a clinical study to evaluate the MAM in clinical practice using the IMIDoc digital health tool. We developed this tool in collaboration with clinicians, biomedical engineers, and patients using a participatory or team-based approach. We believe that close collaboration between health care professionals and patients is essential for developing mHealth solutions tailored to patients' needs.

Digital health solutions or tools based on the use of mHealth technologies can empower patients by supporting self-management skills and providing information on health status and symptom management [8,17,22]. The application of health information technologies and wearable monitoring is widespread in other fields of medicine, such as brain health, HIV, and cancer [23-25]. In addition, mHealth supports the follow-up of symptoms that rheumatologists might miss during time-limited visits, thereby helping reduce the mismatch between issues important to patients versus those important to clinicians [26]. Piga et al [27] published a systematic review of tools for remote monitoring and treatment that included studies of patients with rheumatoid arthritis, systemic sclerosis, fibromyalgia, and osteoarthritis. Their study showed that feasibility and patient satisfaction rates were high or very high depending on the type of intervention and that the efficacy of these interventions was at least similar to that of standard care. Thus, it was shown that telemedicine could assist both rheumatologists in monitoring treatment and patients in participating in their treatment. However, according to the same study, the tools developed were generally unidirectional, serving either the clinician in disease assessment and treatment monitoring or the patient as an educational or disease support tool.

In this context, 2 systematic reviews have shown that there is scant evidence concerning instruments and their use for following up patients with RA [28,29]. Although several projects, such as My Joint Pain or RAHelp [30-32], have shown that telemedicine can provide a new opportunity for managing patients with chronic illness, further research is still needed, as implementation in clinical practice remains very limited [18]. Some new tools have recently been designed ranging from rheumatology referral support [33] to symptom capture [34]; however, the evidence on app use in IMIDs lags far behind that of other fields in terms of treatment adherence [35] or disease symptom improvement [36].

Both the development and implementation of mHealth technologies present several challenges and risks. In this regard, our project meets relevant Sustainable Development Goals adopted by the United Nations. First, our project addresses the goal of “good health and well-being” in the interest of promoting better patient care. Since this project focuses on chronic inflammatory diseases, good disease control can empower patients to reduce their functional disability, resulting in less impact on the quality of life. All this is only possible with a personalized follow-up approach, enabling clinicians to anticipate disease damage. The ability to access more detailed information on patient evolution and adherence problems or adverse effects can ameliorate the repercussions of erroneous therapeutic decisions [37,38].

Overall, this project aims to have both an economic and psychosocial impact on our health care system. On the one hand, the use of these tools can help educate patients about their disease, providing instructions on self-management for certain disease aspects. This can result in fewer visits to the emergency department, fewer consultations, and better therapeutic compliance. On the other hand, the availability of ever-greater amounts of data will make it possible to identify challenges or issues that would otherwise go unnoticed, thus avoiding incorrect strategies. New disease management strategies aimed at more appropriate resource use, based on clinical practice data, are needed.

## Abbreviations

ACPA: anticitrullinated peptide antibodies
ASAS: Assessment of Spondyloarthritis International Society
ASAS-HI: Assessment of Spondyloarthritis International Society Health Index
ASDAS: Ankylosing Spondylitis Disease Activity Score
b/ts-DMARD: biologic or targeted synthetic disease-modifying antirheumatic drug
BASDAI: Bath Ankylosing Spondylitis Disease Activity Index
BASFI: Bath Ankylosing Spondylitis Functional Index
CRP: C-reactive protein
csDMARD: conventional disease-modifying antirheumatic drug
CTU: complex therapy unit
DAS28: Disease Activity Score 28
ePRO: electronic patient-reported outcome
ESR: erythrocyte sedimentation rate
GDPR: General Data Protection Regulation
HAQ: Health Assessment Questionnaire
HLA: histocompatibility antigen
HUB: Hospital Universitario Bellvitge
HUIL: Hospital Universitario Infanta Leonor
HULP: Hospital Universitario La Paz
HURyC: Hospital Universitario Ramón y Cajal
HUVH: Hospital Universitario Vall d´Hebron
IMID: immune-mediated disease
MAM: mixed attention model
mHealth: mobile health
NPS: Net Promoter Score
PtDAS28: patient-derived Disease Activity Score 28
RA: rheumatoid arthritis
RF: rheumatoid factor
RMD: rheumatic and musculoskeletal disease
SDAI: Simplified Disease Activity Index
SJC: swollen joint count
SpA: spondylarthritis
sSJC: self-assessed swollen joint count
sTJC: self-assessed tender joint count
SUS: System Usability Scale
TJC: tender joint count
